# Prolonged hematopoietic and myeloid cellular response in patients after an acute coronary syndrome measured with ^18^F-DPA-714 PET/CT

**DOI:** 10.1007/s00259-018-4038-8

**Published:** 2018-05-04

**Authors:** Simone L. Verweij, Lotte C. A. Stiekema, Ronak Delewi, Kang H. Zheng, Sophie J. Bernelot Moens, Jeffrey Kroon, Charlotte I. Stroes, Miranda Versloot, Jan J. Piek, Hein J. Verberne, Erik S. G. Stroes

**Affiliations:** 1 Department of Vascular Medicine, Academic Medical Center, PO Box 22660, 1100 DD Amsterdam, The Netherlands; 2 Department of Cardiology, Academic Medical Center, PO Box 22660, 1100 DD Amsterdam, The Netherlands; 3 Department of Experimental Vascular Medicine, Academic Medical Center, PO Box 22660, 1100 DD Amsterdam, The Netherlands; 4 Department of Radiology and Nuclear Medicine, Room F2-238, Academic Medical Center, PO Box 22660, 1100 DD Amsterdam, The Netherlands

**Keywords:** Acute coronary syndrome, ^18^F-DPA-714 PET/CT, Hematopoietic organs, Monocytes, Hematopoietic stem and progenitor cells

## Abstract

**Purpose:**

An acute coronary syndrome (ACS) is characterized by a multi-level inflammatory response, comprising activation of bone marrow and spleen accompanied by augmented release of leukocytes into the circulation. The duration of this response after an ACS remains unclear. Here, we assessed the effect of an ACS on the multi-level inflammatory response in patients both acutely and after 3 months.

**Methods:**

We performed ^18^F-DPA-714 PET/CT acutely and 3 months post-ACS in eight patients and eight matched healthy controls. DPA-714, a PET tracer binding the TSPO receptor and highly expressed in myeloid cells, was used to assess hematopoietic activity. We also characterized circulating monocytes and hematopoietic stem and progenitor cells (HSPCs) by flow cytometry in 20 patients acutely and 3 months post-ACS and in 19 healthy controls.

**Results:**

In the acute phase, patients displayed a 1.4-fold and 1.3-fold higher ^18^F-DPA-714 uptake in, respectively, bone marrow (*p* = 0.012) and spleen (*p* = 0.039) compared with healthy controls. This coincided with a 2.4-fold higher number of circulating HSPCs (*p* = 0.001). Three months post-ACS, ^18^F-DPA-714 uptake in bone marrow decreased significantly (*p* = 0.002), but no decrease was observed for ^18^F-DPA-714 uptake in the spleen (*p* = 0.67) nor for the number of circulating HSPCs (*p* = 0.75).

**Conclusions:**

^18^F-DPA-714 PET/CT reveals an ACS- triggered hematopoietic organ activation as initiator of a prolonged cellular inflammatory response beyond 3 months, characterized by a higher number of circulating leukocytes and their precursors. This multi-level inflammatory response may provide an attractive target for novel treatment options aimed at reducing the high recurrence rate post-ACS.

**Electronic supplementary material:**

The online version of this article (10.1007/s00259-018-4038-8) contains supplementary material, which is available to authorized users.

## Introduction

In the first 6–9 months following an acute coronary syndrome (ACS), patients face a disproportionally increased risk of re-infarction [1]. Recent experimental studies suggest a crucial role for a multi-level inflammatory response post-ACS [2]. In murine models, ligation of the coronary artery results in increased bone marrow and spleen activation, leading to augmented release of leukocytes into the circulation [2]. These mobilized leukocytes, predominantly myeloid cells, facilitate the progression of atherosclerosis, contributing to growth as well as destabilization of atherosclerotic plaques [3]. Following an ACS in patients, studies have substantiated that ^18^F-fluordeoxyglucose (^18^F-FDG) uptake in bone-marrow and spleen is increased, visualized with positron emission tomography/ computed tomography (PET/CT). This increased uptake coincides with increased inflammatory parameters in plasma and arterial wall inflammation [4, 5]. Whether this multi-level inflammatory response persists after the acute phase, thereby potentially contributing to the increased re-infarction risk post-ACS, remains unclear.

Although ^18^F-FDG PET/CT imaging is a validated technique for quantifying inflammation in atherosclerotic plaques [6], FDG is a non-specific glucose analogue reflecting a change in overall metabolic activity. In this respect, FDG uptake in bone marrow and spleen may reflect a wide array of cells and processes, ranging from enhanced cellular activation to increased proliferative activity. Thus, more specific imaging tracers are required to further delineate the role of myeloid cells in the multi-level inflammatory response in patients post-ACS. The tracer N,N-diethyl-2-(2-(4-(2-fluoroethoxy)phenyl) 5,7dimethylpyrazolo [1,5a] pyrimidin-3-yl)acetamide (DPA-714) has been widely used for imaging of neuro-inflammatory processes [7–9]. DPA-714 is a second generation tracer for the translocator protein (TSPO) receptor [10], which is expressed predominantly on cells from the myeloid lineage [11], comprising macrophages, monocytes and microglial cells. Since expression of the TSPO receptor does not change in human myeloid cells following inflammatory stimuli [12], TSPO-targeting tracers for PET/CT are more likely to reflect changes in myeloid cell number rather than in activation phenotype. In support, TSPO-PET studies in patients revealed a higher target to background in symptomatic carotid plaques, whereas TSPO-binding was also found to co-localize with the macrophage CD68 marker in excised atherosclerotic plaques [13]. This suggests that the TSPO receptor could be a valuable target for quantifying the number of myeloid cells in bone marrow and spleen using ^18^F-DPA-714 PET/CT.

In the present study, we used ^18^F-DPA-714 PET/CT combined with flow cytometry to investigate the acute and prolonged (3 months and up to 2 years) effects of an ACS on myeloid cellular responses in both hematopoietic organs (bone marrow and spleen) as well as in the plasma compartment (progenitor cell numbers and monocyte phenotypes).

## Material and methods

### Study population and design

We performed a case-control study in 20 ACS patients (50 years or older, ACS documented with electrocardiogram and/or cardiac enzymes). Exclusion criteria were previous cardiovascular events, diabetes mellitus, chronic kidney disease, peripheral artery disease and any chronic inflammatory disease. Blood withdrawal for flow cytometry and the measurement of lipid levels and inflammatory parameters was performed within 3 days and 3 months post-ACS. ^18^F-DPA-714 PET/CT was performed within 10–18 days and also 3 months post-ACS. As controls, 19 healthy subjects matched for age and gender were included. Vital parameters, including weight, height and blood pressure were measured at baseline. Hypertension was defined as the use of antihypertensive medication prior to the event.

The study protocol was approved by the institutional review board of the Academic Medical Center in Amsterdam, the Netherlands and conducted according to the principles of the declaration of Helsinki. Written informed consent was obtained from each participant.

### ^18^F-DPA-714 PET/CT

^18^F-DPA-714 PET/CT was performed on a dedicated scanner (Philips, Best, the Netherlands). DPA-714 (100 MBq) was injected as a bolus lasting 1 min, followed by a redistribution phase of approximately 60 min [8], after which a PET/CT was performed in combination with a low-dose, non-contrast-enhanced CT for attenuation correction and anatomic co-registration [6]. Images were analyzed by experienced readers blinded for patient data, using dedicated software (Hybrid viewer, HERMES medical solutions AB, Stockholm, Sweden). In patients, ^18^F-DPA-714 uptake was assessed in bone marrow and spleen 10–18 days after the event, and repeated after 3 months. Healthy controls underwent an ^18^F-DPA-714 PET/CT scan once. ^18^F-DPA-714 uptake in the bone marrow was assessed by drawing regions of interest (ROIs) in the lumbar vertebrae, and uptake in the spleen was assessed by drawing five ROIs in the axial plane [4]. ^18^F-DPA-714 uptake was determined as the mean of maximal standard uptake value (SUV_max_). We also assessed the corrected SUV of bone marrow and spleen, using DPA-714 uptake in the pectoral muscle as background (DPA-714 uptake in bone marrow or spleen minus DPA-714 uptake in pectoral muscle). Additional information on the DPA-714 tracer can be found in the supplemental methods.

A polymorphism of the TSPO gene is responsible for three different binding affinities of ^18^F-DPA-714 to the TSPO receptor: a high, intermediate and low binding affinity [14]. We only scanned patients with high and intermediate binding affinity and matched the healthy controls for similar binding affinity. Genotyping is described in the supplemental methods.

### Flow cytometry of HSPCs and monocytes

Flow cytometry of circulating HSPCs and monocytes was performed within 3 days post-ACS and repeated 3 and 6–24 months post-ACS. A mononuclear cell fraction (MNC) was isolated using Ficoll; MNCs were stored in medium (RPMI 1640-medium +20% Fetal calf serum +1% penicillin-streptomycine). Cells were incubated with fluorochrome labeled antibodies (supplemental table S1). Samples were analyzed using a FACS Canto-B Tube Loader. HSPCs were classified as CD34^+^CD45_dim_ cells (supplemental Figure S1A-D) [15]. Monocytes were classified according to CD45, CD14 and CD16 expression (supplemental Figure S1E-I), with subsequent determination of surface markers involved in monocyte adhesion and migration (supplemental Table S1). Samples were analyzed using FlowJo software (version 10.0 FlowJO, LLC, Ashland, OR). Delta median fluorescence intensity (MFI) was obtained by subtracting the MFI from an unstained control from the MFI of the marker. Flow cytometry analysis of TSPO receptor expression is provided in the supplemental methods.

### Statistical analysis

Data were analyzed using Prism version 6.0 (GraphPad software, LaJolla, California) and SPSS version 23.0 (SPSS Inc., Chicago, Illinois). Data are presented as the mean ± standard deviation (SD) and median with interquartile range (IQR) for, respectively, normally and non-normally distributed data or as a number (percentage) for categorical variables. Differences in clinical characteristics, number of circulating HSPCs, monocyte phenotype and ^18^F-DPA-714 uptake between patients and healthy controls were assessed using Student’s t-test or Mann Whitney U-test for normally and non-normally distributed variables, respectively. To assess differences between baseline measurements and the repeated measurements, paired Student’s t-tests or Wilcoxon signed rank tests for normally and non-normally data, respectively, were performed. A two-sided *p*-value <0.05 was considered statistically significant.

## Results

### Study population

We included 20 patients with an ACS (63 ± 8 years, 80% male), with troponin levels of 1.3 [0.2–5.5] μg/L, including 16 patients with a ST-Elevation Myocardial Infarction (STEMI) and four patients with a Non-ST Elevation Myocardial Infarction (NSTEMI). Nineteen healthy controls (62 ± 9 years, 63% male) matched for age and gender were also included. Baseline characteristics are listed in Table 1. Additional baseline clinical characteristics of the ACS patients are provided in the supplements (Table S3). Patients with an ACS had an adverse cardiovascular risk profile compared with healthy controls, including a higher body mass index (BMI), a higher prevalence of hypertension and a trend towards more active smokers. Patients had higher HDL levels; other lipid levels were comparable between patients and controls. Patients in the acute phase were characterized by elevated inflammatory parameters (CRP and leukocyte count) compared with healthy controls. After the event, acetyl-salicylic acid and ticagrelor treatment was initiated in all patients; if not used yet, anti-hypertensive medication and/or statin treatment was provided in line with current guidelines. At baseline, troponin levels were associated with plasma monocyte count (*r* = 0.517, *p* = 0.012) with a trend for association with CRP levels (*r* = 0.385, *p* = 0.094).

**Table 1:** Baseline characteristics

Baseline characteristics	ACS patients; acute phase (*n* = 20)	Healthy controls (*n* = 19)	*P*-value acute phase vs healthy controls	Patients; 3 months post-ACS (*n* = 16)	*P*-value acute phase vs 3 months post-ACS	*P*-value 3 months post-ACS vs healthy controls
Age, years	63 ± 8	62 ± 9	0.503	n/a	n/a	n/a
Sex, men/women	16/4	12/7	0.412	n/a	n/a	n/a
BMI, kg/m^2^	28.5 ± 5	25 ± 3	0.026	28.7 ± 5	0.351	0.014
Systolic blood pressure, mmHg	135 ± 18	127 ± 16	0.189	n/a	n/a	n/a
Diastolic blood pressure, mmHg	79 ± 13	80 ± 11	0.696	n/a	n/a	n/a
Smoking, yes/past/no	6/7/7	1/7/11	0.137	n/a	n/a	n/a
Hypertension, yes/no	9/11	0/19	0.001	n/a	n/a	n/a
CRP, mg/dl	13.2[5.2–18]	0.8[0.4–1.2]	<0.001	1.7[0.4–3.7]	0.001	0.042
Total cholesterol, mmol/L	5.1 ± 0.7	5.6 ± 1.0	0.087	3.9 ± 0.7	<0.001	<0.001
HDL cholesterol, mmol/L	1.3 ± 0.5	1.7 ± 0.4	0.014	1.4 ± 0.5	0.667	0.030
LDL cholesterol, mmol/L	3.2 ± 0.8	3.5 ± 0.8	0.295	2.0 ± 0.5	<0.001	<0.001
Triglycerides, mmol/L	1.2[0.8–1.7]	0.8[0.5–1.3]	0.065	1.1[0.8–1.3]	0.887	0.059
Leukocytes, *10^9/L	9.7 ± 2.6	4.9 ± 0.8	<0.001	7.3 ± 1.9	0.012	<0.001
Neutrophils, *10^9/L	6.0 ± 3.1	2.7 ± 0.6	<0.001	4.2 ± 1.1	0.061	<0.001
Lymphocytes, *10^9/L	2.3 ± 0.6	1.55 ± 0.5	<0.001	2.1 ± 0.9	0.603	0.021
Monocytes, *10^9/L	0.9 ± 0.3	0.4 ± 0.1	<0.001	0.7 ± 0.3	0.035	<0.001

Values are *n*, mean ± SD or median [IQR] for normally and non-normally distributed data, respectively

*BMI* body mass index, *CRP* C-reactive protein, *HDL* high-density cholesterol, *LDL* low-density cholesterol

Three months post-ACS, lipid levels were significantly lower, corresponding to the start of statin treatment. CRP levels and leukocyte counts decreased significantly, as shown in Table 1, although these inflammatory parameters remained elevated compared with healthy controls No serious adverse events occurred in the participating ACS patients during the 3-month study period.

### Elevated DPA-714 uptake in bone marrow and spleen

**Fig. 1:**
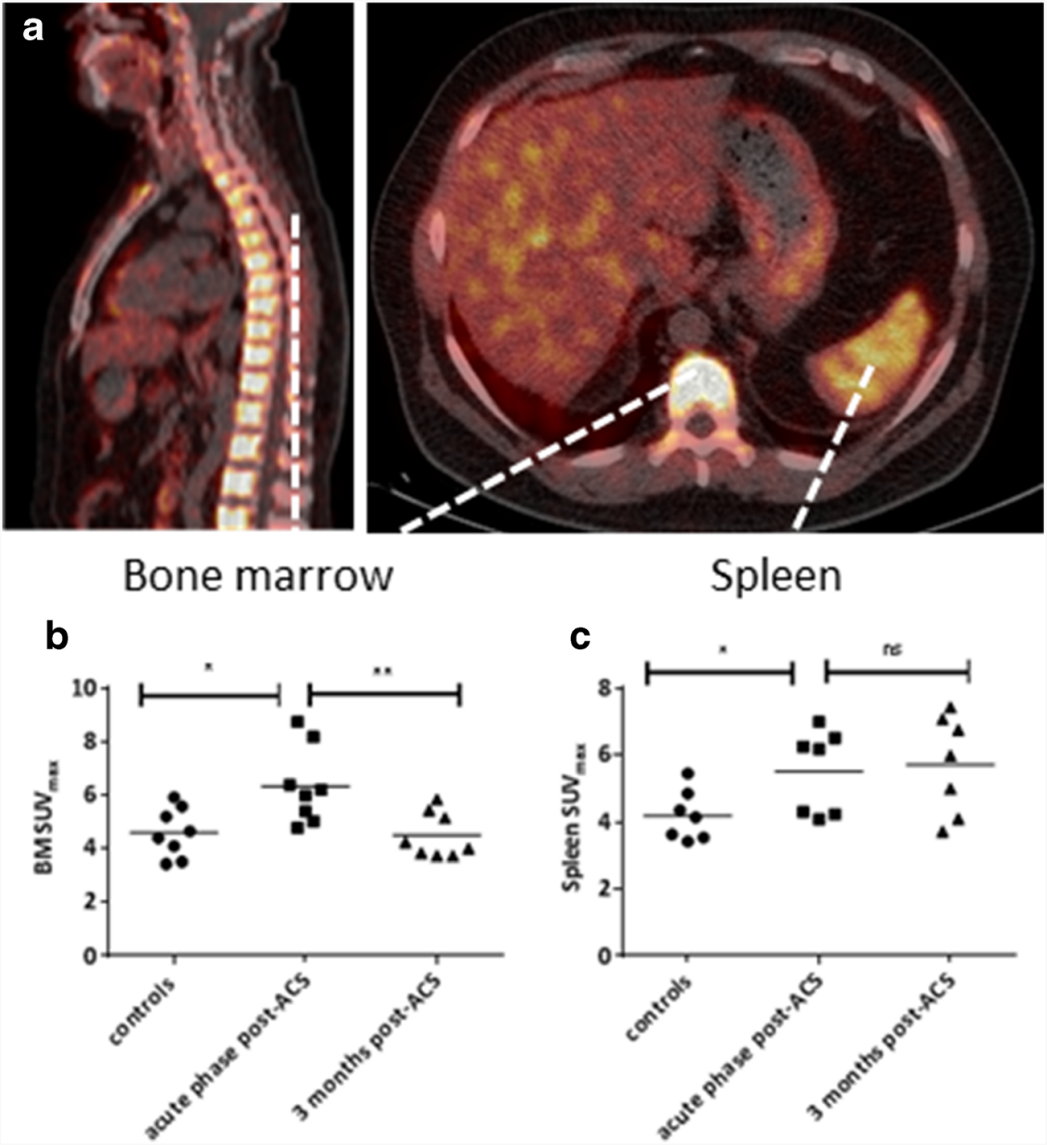
Elevated ^18^F-DPA-714 uptake in bone marrow and spleen post-ACS. ^18^F-DPA-714 uptake in the bone marrow and spleen (*yellow color* in **a**) was quantified as maximal standardized uptake value (SUV_max_). ^18^F-DPA-714 uptake in the bone marrow was assessed by drawing regions of interest (ROIs) in the lumbar vertebrae (visible on both sagittal and transversal views), uptake in the spleen was assessed by drawing five ROIs in the axial plane (visible on the transversal view). Patients in the acute phase post-ACS showed elevated DPA-714 uptake in the bone marrow and spleen compared with healthy controls. Three months post-ACS, ^18^F-DPA-714 uptake in bone marrow decreased (**b**), which no longer differed from healthy controls, while ^18^F-DPA-714 uptake in the spleen remained elevated (**c**), which is still significantly higher compared with healthy controls. Data are represented as mean with single values of the subjects, *p < 0.05, **p < 0.01. *BM *bone marrow, *ns* non-significant, *SUV* standardized uptake value

In eight patients and eight matched controls, we assessed hematopoietic activity using ^18^F-DPA-714 uptake in bone marrow and spleen (for one patient, splenic data was insufficient) (Fig. 1a). In the acute phase, the scan was performed 14 ± 2 days post-ACS. Baseline characteristics of this subgroup were comparable to the whole cohort (Table S4).

In the acute phase, patients with an ACS showed a 1.4-fold higher uptake of ^18^F-DPA-714 in the bone marrow (*p* = 0.012) as well as a 1.3-fold higher uptake in the spleen (*p* = 0.039) compared with healthy controls (Fig. 1b-c). Three months post-ACS, ^18^F-DPA-714 uptake in bone marrow decreased significantly (SUV-BM_max_ acute phase 6.3 ± 1.4 versus 3 months 4.4 ± 0.8, *p* = 0.002), which no longer differed from healthy controls (*p* = 0.813). In contrast, ^18^F-DPA-714 uptake in the spleen remained elevated (acute phase: 5.5 ± 1.2 versus 3 months: 5.7 ± 1.5, *p* = 0.671), which is still significantly higher compared with healthy controls (*p* = 0.032). In the supplements, Figure S2 shows individual lines connecting individual data points (acute phase and after 3 months) per ACS patient. When applying background correction, corrected SUV values show similar results substantiating significant differences between controls and ACS patients in the acute phase (bone marrow: *p* = 0.009; spleen: *p* = 0.028), followed by a significant decrease of bone marrow corrected SUV after 3 months (*p* = 0.0018), at which time point spleen corrected SUV remained elevated (*p* = 0.352) (supplemental Figure S3).

To assess potential cell specific DPA-714 uptake in bone marrow and spleen, we determined the expression of the TSPO receptor on HSPCs and myeloid lineage cells. As shown in supplemental Figure S4, the TSPO receptor is highly expressed on monocytes and less on lymphocytes. Furthermore, the TSPO receptor is also highly expressed on HSPCs.

### Higher number of circulating HSPCs

**Fig. 2:**
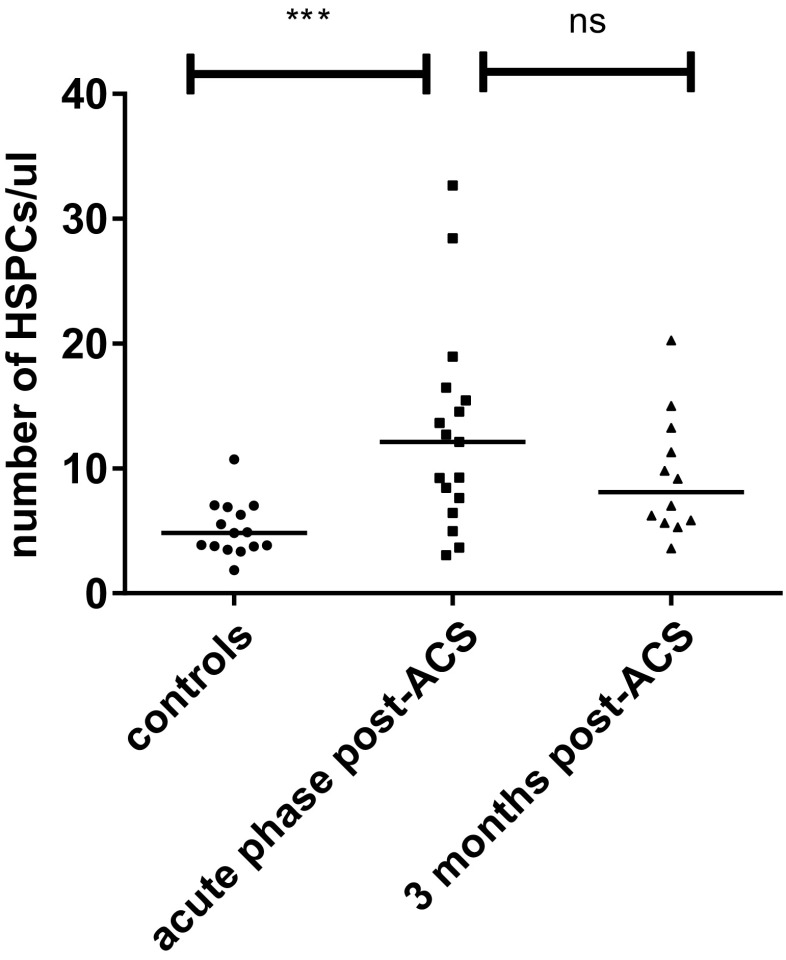
Elevated number of circulating HSPCs post-ACS. The number of circulating HSPCs is assessed using flow cytometry, classifying HSPCs as CD34^+^CD45_dim_ cells. Patients in the acute phase showed significantly elevated numbers of circulating HSPCs compared with healthy controls. Three months post-ACS, there is a numerical decrease in the number of circulating HSPCs; however, the number of circulating HSPCs is still significantly higher compared with healthy controls. Data are represented as median with single values of the subjects, *p < 0.05, **p < 0.01, ***p < 0.001. *HSPCs* hematopoietic stem and progenitor cells, *ns* non-significant

Following the elevated ^18^F-DPA-714 uptake in bone marrow and spleen, we assessed the number of circulating HSPCs in the acute phase post-ACS and after 3 months. Using flow cytometry, we observed that patients in the acute phase had a 2.5-fold higher number of circulating HSPCs compared with healthy controls (patients 12 [7–16] cells/μl versus controls 5 [4–7] cells/μl, *p* = 0.001; Fig. 2). This coincided with a trend towards myeloid skewing of circulating inflammatory cells; there was a significantly lower percentage of lymphoid cells and a trend towards elevated percentages of monocytes (supplemental Table S5).

After 3 months, the number of circulating HSPCs of patients showed a numerical decrease (acute phase 12 [7–16] cells/μl versus 3 months 8 [6–13] cells/μl, *p* = 0.754; Fig. 2). At this point in time, the number of circulating HSPCs was still higher compared with healthy controls (*p* = 0.009). Myeloid skewing of inflammatory cells disappeared (supplemental Table S5).

### Elevated CCR2 expression on monocytes

**Fig. 3:**
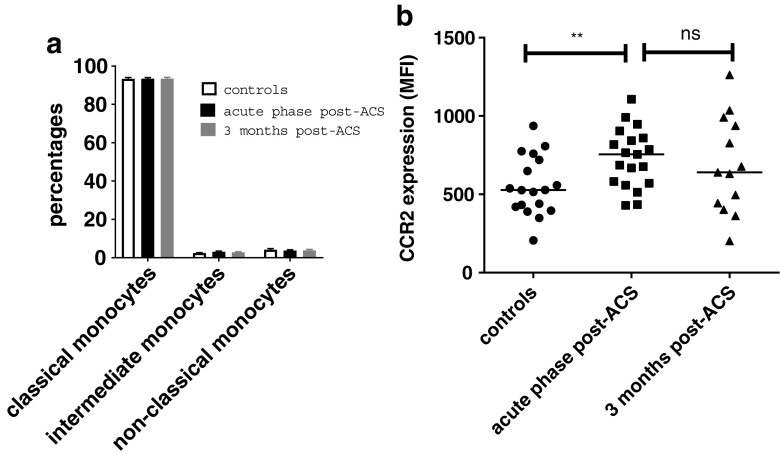
Elevated CCR2 expression on monocytes post-ACS. Monocyte subset distribution, classified according to CD14 and CD16 expression, and CCR2 expression on monocytes were assessed using flow cytometry. Monocyte subset distribution was similar in both the acute phase and 3 months post-ACS. The highest percentage of monocytes consisted of classical monocytes (CD14^+^CD16^−^; 93%), followed by both intermediate (CD14^+^CD16^+^; 3%) and non-classical monocytes (CD14^+^CD16^++^; 4%) in almost equal percentages (**a**). However, in the acute phase we showed an elevated expression of CCR2 on monocytes, with a non-significant decrease after 3 months post-ACS (**b**), which now no longer differed from healthy controls. Data are presented for A as mean ± sem and as median with single values of the subjects for B, *p < 0.05, **p < 0.01. *ACS *acute coronary syndrome

Previous data suggest that in addition to increased cell counts, leukocytes/monocytes may show a more pro-inflammatory phenotype post-ACS [2]. Using flow cytometry, we observed that the distribution of the monocyte subsets (divided in classical (CD14^+^CD16^−^), intermediate (CD14^+^CD16^+^) and non-classical monocytes (CD14^+^CD16^++^)) in the circulation were similar in both the acute phase and 3 months post-ACS, comparable with the distribution of monocyte subsets in healthy controls (Fig. 3a). Interestingly, monocyte expression of the chemokine receptor CCR2, involved in migration of monocytes into the arterial wall, was significantly elevated directly post-ACS compared with healthy controls (CCR2 expression acute phase: 755 [570–860] MFI versus controls 528 [414–729] MFI, *p* = 0.006). Three months post-ACS, the expression of CCR2 showed a numerical decrease (CCR2 expression 636 [393–951] MFI, *p* = 0.36; Fig. 3b), which now no longer differed from healthy controls (*p* = 0.44).

### Long-term cellular response post-ACS

In view of the prolonged inflammatory response after 3 months, we explored persistence up to 6–24 months post-ACS. Flow cytometry on HSPCs and monocytes was repeated in 12 patients, on average 19 [10–21] months post-ACS, and compared with the 3-month time point. Inflammatory parameters are listed in supplemental Table S6. Our data showed a trend towards a decrease of leukocytes 6–24 months post-ACS. Other inflammatory parameters as well as the number of circulating HSPCs were comparable to the level of the 3-month time point (*p* = 0.4; supplemental Figure S5A), despite the use of guideline-based CV-therapy in these patients. However, the activation of circulating monocytes 6–24 months post-ACS, based on their CCR2 expression, showed a trend towards a decrease compared with the level of the 3-month time point (*p* = 0.06), back to levels observed in healthy controls (supplemental Figure S5B). No serious adverse events occurred in the subgroup of 12 patients with a follow-up of 6–24 months.

## Discussion

In the present study, we observed elevated ^18^F-DPA-714 uptake in bone marrow and spleen compared with controls in the acute phase post-ACS, coinciding with a higher number of circulating HSPCs as well as an increased monocyte count showing a pro-inflammatory phenotype. Three months post-ACS, ^18^F-DPA-714 uptake remained elevated in the spleen only, with a concomitant plasma monocytosis and persistent elevation of circulating HSPCs. Collectively, these data support the presence of a prolonged, multi-level inflammatory response post-ACS.

### Prolonged multi-level inflammatory response post-ACS

Following an ACS, monocytes are important for repair of the damaged heart. In this study, we support the presence of an acute hematopoietic response post-ACS [16], resulting in multi-level inflammatory activation. In the acute phase we observe a leukocytosis and monocytosis, with concomitant elevation of circulating HSPCs [17]. This increased myeloid cellular response in plasma corresponds with an elevated ^18^F-DPA-714 uptake in the bone marrow and spleen compared with healthy controls. Using flow cytometry, we substantiated the expression of the TSPO receptor particularly on monocytes and HSPCs, supporting the concept that DPA-714 uptake occurs predominantly in myeloid cells and HSPCs in bone marrow and spleen. Therefore, we used the ^18^F-DPA-714 signal as a reflection of the number of myeloid cells and HSPCs in the hematopoietic organs [12].

Three months post-ACS, ^18^F-DPA-714 uptake in bone marrow was no longer significantly different from healthy controls, which implies a transient increase of cellular density in the bone marrow. In contrast, we recently reported a persistently elevated metabolic activity in bone marrow in patients ≥1 year after a CV-event, using ^18^F-FDG PET/CT [18]. This apparent discrepancy may relate to the different molecular targets of DPA-714 and FDG. Whereas DPA-714 reflects predominantly myeloid cell number [12], FDG reflects overall metabolic activity. Combined, these data imply normalization of myeloid cell count in the bone marrow 3 months post-ACS, whereas overall metabolic activity of the bone marrow may persist for a longer time.

In contrast to the transient elevation of ^18^F-DPA-714 uptake in bone marrow, uptake in the spleen remained elevated 3 months post-ACS, with a concomitant monocytosis and persistent elevation of circulating HSPCs. Whereas the bone marrow is the main organ for hematopoiesis in humans, it has been shown previously that the spleen can facilitate extramedullary hematopoiesis [19]. The latter corresponds with observations in experimental models, showing that a CV-event results in a marked release of HSPCs from the bone marrow to the spleen followed by prolonged extramedullary hematopoiesis in this organ [2, 20]. In mice, increased numbers of circulating HSPCs and monocytes in the spleen were also still present 3 months after the acute event [2], which corresponds to our finding in post-ACS patients.

### Transient monocyte activation post-ACS

Even after 6–24 months post-ACS, HSPCs and monocyte counts in the circulation remained elevated compared with matched controls. In the acute phase, the increased monocyte count was found to be accompanied by monocyte activation, illustrated by increased expression of CCR2. CCR2 is the major chemokine receptor contributing to migration of monocytes into the arterial wall. We recently reported a close correlation between CCR2 expression on circulating monocytes and transendothelial migration ex vivo [21]. More recently, we also observed a correlation between monocyte CCR2 expression and arterial wall inflammation in patients at increased CV-risk [22]. Collectively, these findings support the concept that increased monocyte count, showing an activated phenotype, may partly contribute to the elevated recurrence risk in the first 6 months post-ACS.

### Study limitations

This study has several limitations. First, PET/CT could only be performed in a relatively limited number of subjects. The latter precludes adjustment for potential confounders and correlation with infarct size. Notwithstanding, we did observe significant differences in ^18^F-DPA-714 uptake, comparable to studies using ^18^F-FDG PET/CT in the acute phase post-ACS. Second, we compared ACS patients in the acute phase with healthy controls, followed by a repeat scan after 3 months. In absence of a scan preceding the event, we cannot exclude that the ACS patients may already have elevated hematopoietic activity prior to the acute event [4]. However, DPA-714 uptake in bone marrow was no longer different in ACS patients compared with matched controls 3 months post-ACS, which suggests an event-related ‘transient’ increased uptake. Third, TSPO binding is significantly affected by polymorphisms of the TSPO gene. To minimize potential impact of these polymorphisms, we excluded the low-binding affinity genotype and matched patients and controls for intermediate or high binding affinity. Finally, the TSPO receptor is also expressed in various peripheral tissues in humans [10]. In the heart and arterial wall, we observed a continuous high signal for DPA-714 in controls as well as patients with or without a recent event. Since the signals in these organs were comparably high directly following an ischemic event and after 3 months, this most likely represents non-specific binding of DPA-714 to smooth muscle cells [10]. This data shows that DPA-714 performs worse than the more traditional FDG as a tracer to measure inflammatory activity in cardiovascular organs [6].

## Conclusions

^18^F-DPA-714 PET/CT showed an ACS-triggered hematopoietic organ activation as initiator of a prolonged leukocyte oversupply, characterized by an elevated number of circulating leukocytes and their precursors. Further studies are required to determine whether strategies aimed at reducing this prolonged hematopoietic activation may translate into a reduced re-infarction rate in the vulnerable post-ACS period.

## Electronic supplementary material

ESM 1 (DOC 1966 kb)
