# Supplementary material for: Prolonged hematopoietic and myeloid cellular response in patients after an acute coronary syndrome measured with 18F-DPA-714 PET/CT
Source: Eur J Nucl Med Mol Imaging. 2018 May 4;45(11):1956–63. doi: 10.1007/s00259-018-4038-8 (PMC6132543; doi:10.1007/s00259-018-4038-8)
Supplement: Supplementary file 1 — (DOC 1966 kb) [file 259_2018_4038_MOESM1_ESM.doc]

**SUPPLEMENTAL METHODS**

**Additional information on the tracer DPA-714**

A putative antagonist of the TSPO receptor, DPA-713, has been described previously[1, 2]. Concurrently, the fluoro-ethoxy derivative DPA-714, a TSPO agonist, was developed and labeled with fluorine-18[3]. Both DPA-713 and DPA-714 exhibit higher affinity for the TSPO in vitro (Ki=4.7 nM and Ki=7.0 nM, respectively) than PK11195 (Ki=9.3 nM)[1, 3].

The effective dose of ^18^F-DPA-714 estimated from biodistribution in mice was 17.2 μSv/MBq. Modeling of regional brain and plasma data showed good in vivo stability of ^18^F-DPA-714 in humans, with only 20% of blood metabolites 20 min post-injection . Whole-body images demonstrate uptake in the gallbladder, heart, spleen and kidneys[4].

**Genotyping polymorphism rs6971**
All individuals were genotyped for the rs6971 polymorphism within the TSPO gene. First, DNA was isolated using the QIAamp DNA mini kit 50 (Qiagen, Valencia, California; cat no 51303).Then a qPCR was performed. Per well, 10 ul mastermix (5 ul Taqman Genotyping mastermix (Applies Biosystems, Foster City, USA), 0.1ul primer (rs6971; TSPO gene, thermofisher scientific) and 4.9 ul milliQ) and 10-20 ng DNA was added. Samples were analyzed using Biorad CFX manager.
The genetic analysis showed the binding affinity (high, intermediate or low) for the TSPO receptor per patient.

**TSPO receptor analysis on leukocytes and HSPCs**
The expression of the TSPO receptor on leukocytes and HSPCs was assessed using flow cytometry. For the leukocytes, red blood cells were lysed with red blood cell lysis buffer (Affymetrix, eBioscience, San Diego, USA). Leukocytes were incubated with fluorochrome labelled antibodies (supplemental table S2) for 15 minutes and washed with phosphate buffered saline (PBS). Samples were analyzed using BD FACS Canto II (Becton, Dickinson, Fanklin Lakes, New Jersey). Monocytes were classified according to HLA-DR, CD14 and CD16 expression, B-cells according to CD19 expression, NK cells according to CD56 expression and T cells according to CD3 and CD8 expression. Subsequently, the expression of the TSPO receptor was determined (supplemental table S2). Samples were analyzed using FlowJo software (version 10.0 FlowJO, LLC, Ashland, OR). Delta median fluorescence intensity (MFI) was obtained by subtracting the MFI from an unstained control from the MFI of the marker.
For the HSPCs, sorted CD34+ cells were used. These cells were incubated with a fluorochrome labelled antibody for the TSPO receptor (supplemental table S2). Samples were analyzed using FlowJo software (version 10.0 FlowJO, LLC, Ashland, OR). Delta median fluorescence intensity (MFI) was obtained by subtracting the MFI from an unstained control from the MFI of the marker.

**References**1. Boutin H, Chauveau F, Thominiaux C, Gregoire MC, James ML, Trebossen R et al. 11C-DPA-713: a novel peripheral benzodiazepine receptor PET ligand for in vivo imaging of neuroinflammation. J Nucl Med. 2007;48(4):573-81.
2. Endres CJ, Pomper MG, James M, Uzuner O, Hammoud DA, Watkins CC et al. Initial evaluation of 11C-DPA-713, a novel TSPO PET ligand, in humans. J Nucl Med. 2009;50(8):1276-82. doi:10.2967/jnumed.109.062265.
3. James ML, Fulton RR, Vercoullie J, Henderson DJ, Garreau L, Chalon S et al. DPA-714, a new translocator protein-specific ligand: synthesis, radiofluorination, and pharmacologic characterization. J Nucl Med. 2008;49(5):814-22. doi:10.2967/jnumed.107.046151.
4. Arlicot N, Vercouillie J, Ribeiro MJ, Tauber C, Venel Y, Baulieu JL et al. Initial evaluation in healthy humans of [ 18F]DPA-714, a potential PET biomarker for neuroinflammation. Nuclear Medicine and Biology. 2012;39:570-8. doi:10.1016/j.nucmedbio.2011.10.012.

**SUPPLEMENTAL RESULTS**

**Table S1** Markers used for flow cytometry of circulating HSPCs and monocytes

|  | **Surface markers** | **Color** | **Company** |
| --- | --- | --- | --- |
| **HSPCs** | CD34 | PeCy7 | Beckman Coulter |
|  | CD45 | PacB | BD Horizon |
| **Monocytes** | CD45 | PacB | DAKO |
|  | CD14 | PerCP | BD Pharmingen |
|  | CD16 | APC H7 | BD Pharmingen |
|  | CCR2 | APC | BD Pharmingen |

*APC indicates allophycocyanin; Cy: CyChrome ; FITC: fluorescein isiothiocyanate ; PE: phycoerythrin; PerCP: peridinin-chlorophyll-protein*

**Table S2** Markers used for flow cytometry of the TSPO receptor on circulating leukocytes and HSPCs

|  | **Surface markers** | **Color** | **Company** |
| --- | --- | --- | --- |
| **TSPO receptor** | Ab199779 (anti-PBR antibody) | FITC | Abcam |
| **Monocytes** | HLA-DR | Percp-C5.5 | BD Pharmingen |
|  | CD14 | PEcy7 | BD Pharmingen |
|  | CD16 | APC-Cy7 | BD Pharmingen |
| **B-cells** | CD19 | APC | BD Pharmingen |
| **NK-cells** | CD56 | APC | BD Pharmingen |
| **T-cells** | CD3 | Percp | BD Pharmingen |
|  | CD8 | APC | BD Pharmingen |

*APC indicates allophycocyanin; Cy: CyChrome ; FITC: fluorescein isiothiocyanate ; PE: phycoerythrin; PerCP: peridinin-chlorophyll-protein*

**Table S3** Additional baseline characteristics ACS patients

| **Characteristics** | **ACS patients (n=20)** |
| --- | --- |
| Troponin levels, ug/L | 1.340 [0.215 – 5.245] |
| Infarct region |  |
| - anterior infarction, n (%) | 14 (70%) |
| - inferior infarction, n (%) | 3 (15%) |
| - lateral infarction, n (%) | 3 (15%) |
| Type of infarction (STEMI/non-STEMI) | 16/4 |
| LVEF after myocardial infarction |  |
| - Good LVEF (≥55%), n (%) | 7 (35%) |
| - Moderate LVEF (35-55%), n (%) | 2 (10%) |
| - Unknown | 11 (55%) |
| ACS treatment |  |
| - PCI with stent | 19 (95%) |
| - Drug therapy | 1 (5%) |
| Medication use before ACS |  |
| - Diuretics, n (%) | 4 (20%) |
| - ACE inhibitor, n (%) | 2 (10%) |
| - CCB, n (%) | 3 (15%) |
| - β-blocker, n (%) | 4 (20%) |
| - ARB, n (%) | 3 (15%) |
| - Statins, n (%) | 3 (15%) |
| - Anti-inflammatory compounds, n (%) | 0 (0%) |
| Medication use after ACS |  |
| - Diuretics, n (%) | 3 (15%) |
| - ACE inhibitor, n (%) | 12 (60%) |
| - CCB, n (%) | 1 (5%) |
| - β-blocker, n (%) | 15 (75%) |
| - ARB, n (%) | 3 (15%) |
| - Statins, n (%) | 19 (95%) |
| - Anti-inflammatory compounds, n (%) | 0 (0%) |
|  |  |

Values are n (%) or median [IQR]. The normal range for the troponin assay in our institution is 0 – 0,05ug/L.
*ACE indicated Angiotensin Converting Enzyme inhibitor; ACS: Acute Coronary Syndrome; ARB: Angiotensin II Receptor Blocker; CCB: Calcium Channel Blockers; LVEF: Left Ventricular Ejection Fraction; non-STEMI: non-ST Elevated Myocardial Infarction; PCI: Percutaneous Coronary Intervention; STEMI: ST Elevated Myocardial Infarction*

**Table S4** Baseline characteristics of the groups who underwent DPA-714 PET/CT

| **Baseline characteristics** | **ACS patients; acute phase (n=8)** | **Healthy controls (n=8)** | **p value acute phase vs healthy controls** |
| --- | --- | --- | --- |
| Age, years | 62±5 | 61±7 | 0.832 |
| Sex, men/women | 7/1 | 5/3 | 0.281 |
| BMI, kg/m^2^ | 31±6 | 24±2 | 0.011 |
| Systolic blood pressure, mmHg | 127±21 | 122±19 | 0.613 |
| Diastolic blood pressure, mmHg | 71±14 | 76±9 | 0.397 |
| Smoking, yes/past/no | 4/2/2 | 1/4/3 | 0.264 |
| Hypertension, yes/no | 3/5 | 0/8 | 0.055 |
| Statin use, yes/no | 0/8 | 0/8 | 1.0 |
| TSPO receptor mutation, homozygotes / heterozygotes | 3/5 | 3/5 | 1.0 |

Values are n or mean ± SD. *BMI indicates body mass index.*

**Table S5 Myeloid skewing of circulating inflammatory cells post-ACS**

| **Inflammatory cells** | **ACS patients;  acute phase (n=20)** | **Healthy controls (n=19)** | **p-value acute phase vs healthy controls** | **ACS patients; 3 months post-ACS (n=16)** | **p-value acute phase versus 3 months post-ACS** |
| --- | --- | --- | --- | --- | --- |
| Neutrophils, percentages | 60±18% | 56±8% | 0.360 | 58±7% | 0.243 |
| Lymphocytes, percentages | 25±8% | 32±8% | 0.023 | 30±6% | 0.042 |
| Monocytes, percentages | 10±4% | 8±1% | 0.059 | 9%±2 | 0.093 |

Values are percentages, mean ± SD

**Table S6** Inflammatory parameters 6 to 24 months post-ACS

| **Characteristics** | **ACS patients; 3 months post-ACS (n=12)** | **ACS patients; 6 to 24 months post-ACS (n=12)** | **p-value** |
| --- | --- | --- | --- |
| Total cholesterol, mmol/L | 3.9±0.7 | 3.8±0.6 | 0.351 |
| HDL cholesterol, mmol/L | 1.5±0.4 | 1.6±0.4 | 0.088 |
| LDL cholesterol, mmol/L | 2.0±0.5 | 1.8±0.4 | 0.066 |
| Triglycerides, mmol/L | 1.1[0.8-1.3] | 0.9[0.7-1.3] | 0.433 |
| CRP, mg/dl | 1.4[0.3-2.6] | 1.6[0.5-3.3] | 0.721 |
| Leukocytes, *10^9/L | 6.9±1.6 | 6.4±1.2 | 0.080 |
| Neutrophils, *10^9/L | 3.9±1.0 | 3.4±0.8 | 0.010 |
| Lymphocytes, *10^9/L | 2.0±0.8 | 2.0±0.6 | 0.982 |
| Monocytes, *109/L | 0.6±0.1 | 0.6±0.2 | 0.357 |

Values are n, mean ± SD or median [IQR] for respectively normally and non-normally distributed data. *CRP indicates C-reactive protein, HDL; high-density cholesterol, LDL; low-density cholesterol*

**Figure S1** Gating strategy for HSPCs and monocytes using flow cytometry

Flow cytometry on whole blood was performed to study HSPC’s and monocyte subsets and specific surface expression markers. First, leukocytes were separated using forward/sideward scatter (**A**), followed by single cell analysis (**B**), HSPCs were classified as CD34^+^ cells (**C**) and CD45_dim_ cells (**D**).
Regarding the monocyte gating strategy, first single cell leukocytes were separated using forward/sideward scatter (**E, F**). Next, monocytes were classified according to CD45 (**G**) followed by CD14 expression (**H)**. Monocyte subsets were divided according to CD14 and CD16 expression, identifying classical (CD14^+^CD16^-^), intermediate (CD14^+^CD16^+^) and non-classical (CD14^+^CD16^++^) monocytes (**I**).

**
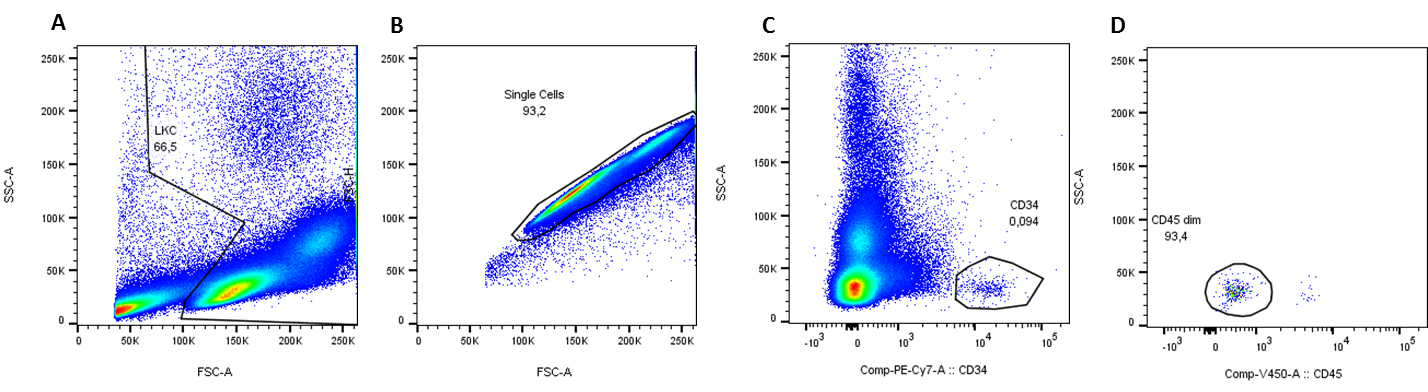

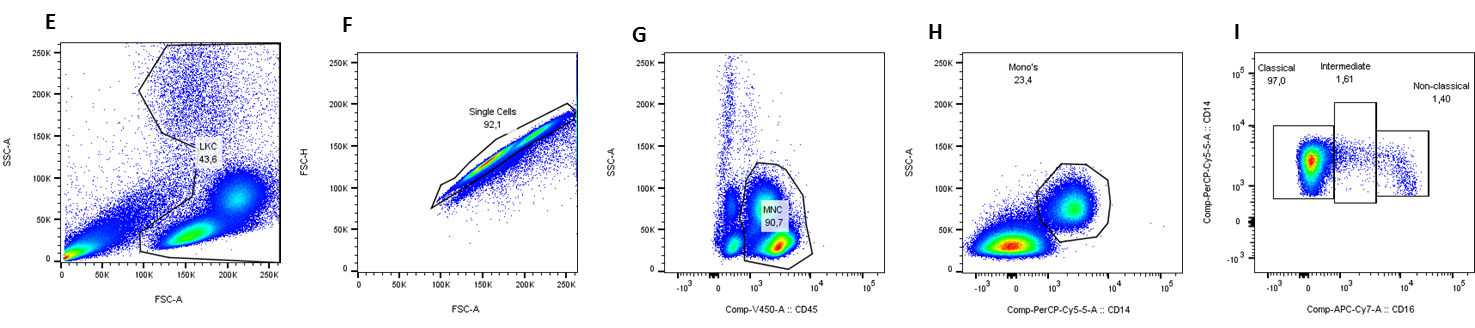
**

**Figure S2** DPA-714 uptake in the acute phase and after 3 months in ACS patients

This figure shows lines connecting individual ^18^F-DPA-714 uptake in bone marrow (**A**) and spleen (**B**) (acute phase and after 3 months) per ACS patient.
*BM indicates bone marrow*

**Figure S3** Elevated ^18^F-DPA-714 uptake in bone marrow and spleen post-ACS shown as corrected SUV

^18^F-DPA-714 uptake in bone marrow and spleen is shown as corrected SUV. Patients in the acute phase post-ACS showed elevated DPA-714 uptake in the bone marrow and spleen compared with healthy controls. Three months post-ACS, ^18^F-DPA-714 uptake in bone marrow decreased (**A**), while ^18^F-DPA-714 uptake in the spleen remained elevated (**B**). Data are represented as mean with single values of the subjects, *p<0.05, **p<0.01
*BM: bone marrow; ns: non-significant*

**Figure S4** Expression of the TSPO receptor on HSPCs and leukocytes

Flow cytometry was performed to study the expression of the TSPO receptor on leukocytes (whole blood) and HSPCs (sorted CD34^+^ cells). As shown in this figure, the TSPO receptor is highly expressed on monocytes and less expressed on the lymphocytes (B-cells, T-cells and NK cells), the TSPO receptor is also relatively high expressed on HSPCs.
*HSPCs indicated hematopoietic stem and progenitor cells; NK: natural killer cells*

**Figure S5** HSPCs and monocytes 6 to 24 months post-ACS

The number of circulating HSPCs and the expression of CCR2 on monocytes (showed as MFI) were assessed using flow-cytometry, comparing the level of the 3 month time point versus 6 to 24 months post-ACS.
6 to 24 months post-ACS, the number of circulating HSPCs were comparable to the numbers of the level of the 3 month time point (**A**; p=0.43), while a trend towards a decrease of CCR2 expression on monocytes 6 to 24 months post-ACS was observed (**B**; p=0.06) using Wilcoxon signed-rank test.

Data are represented as median±IQR
*ACS indicates acute coronary syndrome; HSPCs: hematopoietic stem and progenitor cells*
